# A highly potent human antibody neutralizes dengue virus serotype 3 by binding across three surface proteins

**DOI:** 10.1038/ncomms7341

**Published:** 2015-02-20

**Authors:** Guntur Fibriansah, Joanne L. Tan, Scott A. Smith, Ruklanthi de Alwis, Thiam-Seng Ng, Victor A. Kostyuchenko, Ramesh S. Jadi, Petra Kukkaro, Aravinda M. de Silva, James E. Crowe, Shee-Mei Lok

**Affiliations:** 1 Program in Emerging Infectious Diseases, Duke–NUS Graduate Medical School, 8 College Road, Singapore 169857, Singapore; 2 Centre for BioImaging Sciences, National University of Singapore, 14 Science Drive 4, Singapore 117557, Singapore; 3 Department of Medicine, Vanderbilt University, 1161 21st Avenue South, D-3100 Medical Center North, Nashville, Tennessee 37232-2358, USA; 4 The Vanderbilt Vaccine Center, Vanderbilt University,Vanderbilt University Medical Center, 11475 MRB IV—2213 Garland Avenue, Nashville, Tennessee 37232-0417, USA; 5 Department of Microbiology and Immunology, University of North Carolina at Chapel Hill, CB#7292, 9024 Burnett Womack, Chapel Hill, North Carolina 27599-7292, USA; 6 Departments of Pediatrics and Pathology, Microbiology and Immunology, Vanderbilt University, Vanderbilt University Medical Center, 11475 MRB IV—2213 Garland Avenue, Nashville, Tennessee 37232-0417, USA

## Abstract

Dengue virus (DENV) infects ~400 million people annually. There is no licensed vaccine or therapeutic drug. Only a small fraction of the total DENV-specific antibodies in a naturally occurring dengue infection consists of highly neutralizing antibodies. Here we show that the DENV-specific human monoclonal antibody 5J7 is exceptionally potent, neutralizing 50% of virus at nanogram-range antibody concentration. The 9 Å resolution cryo-electron microscopy structure of the Fab 5J7–DENV complex shows that a single Fab molecule binds across three envelope proteins and engages three functionally important domains, each from a different envelope protein. These domains are critical for receptor binding and fusion to the endosomal membrane. The ability to bind to multiple domains allows the antibody to fully coat the virus surface with only 60 copies of Fab, that is, half the amount compared with other potent antibodies. Our study reveals a highly efficient and unusual mechanism of molecular recognition by an antibody.

## Abstract (web summary)

There is no licensed vaccine or therapeutic for dengue virus (DENV) infection. Here, the authors show that a highly potent human monoclonal antibody binds to DENV particles in an unusual and very effective way by interacting with three viral envelope proteins.

## Graphical abstract

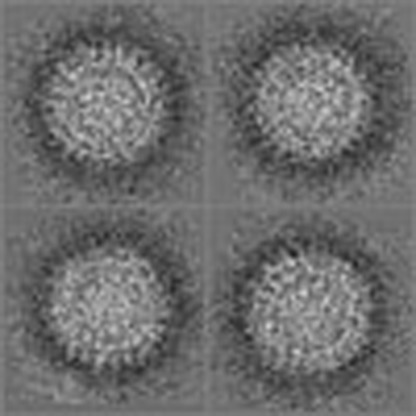

The global incidence of dengue virus (DENV) infection has increased drastically in recent decades. It is estimated that about 400 million people worldwide are infected with DENV annually, resulting in ~100 million cases of dengue fever and 21,000 deaths^1^,^2^. DENV are mainly detected in the tropical and sub-tropical regions around the world, with high incidence reported from the Americas, Eastern Mediterranean, Southeast Asia and the Western Pacific regions. DENV is one of the most important arthropod-borne virus that targets humans. It is transmitted to humans by the bite of infected *Aedes aegypti* or, less frequently, *Aedes albopictus* mosquitoes^3^.

DENV belongs to the Flaviviridae family, along with other major human pathogens such as West Nile virus, Japanese encephalitis virus and yellow fever virus. There are four DENV serotypes (DENV1–4)^4^,^5^. Patients infected with any one of the four serotypes can display a spectrum of symptoms, ranging from being asymptomatic to showing mild dengue fever, to the severe dengue haemorrhagic fever or dengue shock syndrome^6^. An initial primary infection by a DENV serotype induces life-long protection against the homologous serotype^7^. However, in a secondary infection by a different DENV serotype, the formation of non-neutralizing complex of DENV with cross-reactive antibodies from the previous infection may enhance viral infection through a mechanism known as antibody-dependent enhancement^8^. This may lead to an increased risk of developing the severe dengue haemorrhagic fever. This suggests that a safe and effective vaccine would have to include only neutralizing epitopes from all four DENV serotypes. Therefore, mapping of these sites on E protein, recognized by highly neutralizing human antibodies, is crucial for vaccine development.

Inside the DENV particle lies the 11-kb single-stranded positive sense RNA genome complexed with capsid protein. The nucleocapsid is surrounded by a bilayer lipid membrane and on the outside of the membrane are the 180 pairs of envelope (E) and membrane (M) proteins^9^,^10^. The E and M proteins are arranged with icosahedral symmetry with each asymmetric unit containing three pairs of E and M heterodimers. The E proteins exist as head-to-tail homodimers. Three of these dimers lie parallel to each other forming a raft^9^,^10^,^11^. The E protein ectodomain consists of three domains: DI, DII and DIII^12^,^13^,^14^. E protein plays an important role in virus entry into host cell as it binds to receptors and facilitates fusion of the virus to the endosomal membrane^15^,^16^,^17^. Neutralizing antibodies principally target the E protein^18^.

Mouse monoclonal antibody (MAb) studies showed that the most potent antibodies bind to DIII^19^,^20^,^21^, whereas in humans very few antibodies are directed to this region^22^,^23^,^24^,^25^. In a naturally occurring primary dengue infection, a large fraction of the antibody repertoire consists of cross-reactive and poorly neutralizing antibodies, with only a small fraction showing serotype-specific and highly neutralizing properties^26^,^27^,^28^. The highly neutralizing serotype-specific human MAbs (HMAbs) generally recognize quaternary structure-dependent epitopes on the virus surface^26^,^29^.

Here we show that HMAb 5J7 is a very potent antibody that can neutralize DENV3 at nanogram-range concentrations. We determine the cryo-electron microscopy (cryo-EM) structure of DENV3 complexed with Fab 5J7 to 9 Å resolution and show that one Fab molecule binds across three E proteins and engages only domains that are critical for infection. This mode of binding has not been observed previously in any virus system and the results showed the structural basis of neutralization by extremely potent antibodies.

## Results

### HMAb 5J7 prevents virus attachment

**Figure 1:**
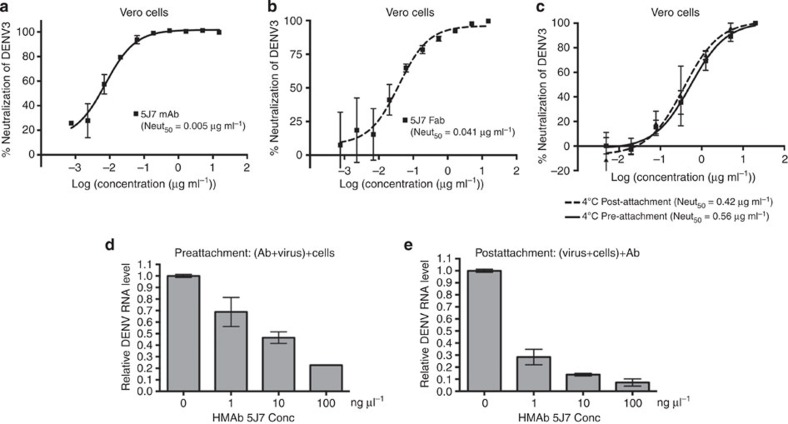
Neutralization of DENV3 infection by HMAb 5J7 in Vero cells. Neut_50_ value of (**a**) HMAb 5J7 and (**b**) its Fab fragment to DENV3 in Vero cells was 0.005 and 0.041 μg ml^−1^, respectively. Fab fragment, although strongly neutralizing, required ~8.2 times higher concentration for neutralization compared with the whole antibody. (**c**) HMAb 5J7 had similar neutralization activities when exposed to virus before or after attachment to Vero cells. Amount of virus on the cell surface, as detected by RT–PCR, when exposed to antibodies (**d**) before and (**e**) after the virus was allowed to attach to cells. The result showed that high concentrations of antibodies are required to prevent attachment of virus to cell surface when virus is exposed to HMAb 5J7 before cell attachment (**d**). On the other hand, HMAb 5J7 seemed to be able to strip the virus off the cell surface efficiently when the virus is exposed to the antibodies post attachment (**e**). Values are mean±s.d. Experiments were repeated at least twice.

We have previously shown that HMab 5J7 exhibited cross-reactive binding to all four DENV serotypes in an enzyme-linked immunosorbent assay (ELISA) and yet strongly neutralized only DENV3 virions^26^,^30^. To provide further support to the type 3-specific binding pattern and the potency of HMAb 5J7, we repeated the binding assays here with intact particles or recombinant E (rE) proteins, and also the neutralization assays as an independent laboratory. The assays showed that HMAb 5J7 is a highly potent DENV3-specific HMAb (50% neutralization concentration [neut_50_] value of 0.005 μg ml^−1^) (Fig. 1a and Supplementary Fig. 1). HMAb 5J7 binds the whole DENV particle at a much higher affinity than the soluble form of the rE protein expressed in insect cells (Supplementary Fig. 1). The Fab fragment is also strongly neutralizing (neut_50_=0.041 μg ml^−1^) (Fig. 1b), although eightfold poorer than the full-length antibody. The higher potency of the whole antibody compared with Fab fragment could be due to several reasons, including (1) higher avidity of the bivalent form of antibody, (2) the ability of the bivalent antibody to aggregate virus particles^31^, or (3) increased steric hindrance of virus–host interactions (such as receptor binding or interaction of virus with the endosomal membrane during fusion) due to the larger size of the whole antibody.

The most common way to determine whether the antibody inhibits virus-receptor binding or a postattachment step of the infection is to compare neutralization curves when antibody is mixed with the virus before or after virus binding to cells at 4 °C (Fig. 1c). The assumption is antibodies that inhibit receptor binding will not have a neutralizing effect on virus that has already bound to its receptor. However, this assumption may not be true, as high-affinity antibodies may displace the virus that is already complexed to a low-affinity receptor. To test this, we used real-time reverse transcriptase–PCR (RT–PCR) to quantify the remaining virus on the cell surface, which were exposed to antibodies pre- and postvirus attachment to cells. The plaque number of pre-and postattachment neutralization tests showed no difference (Fig. 1c); this would suggest that the antibody inhibit a postattachment step of the infection. On the contrary, the RT–PCR results (Fig. 1e) suggest that the antibody displaced the virus that has already bound to the cell receptor, therefore emphasizing that deriving the mechanism of neutralization by just conducting the neutralization tests alone would be misleading. RT–PCR result showed that the virus exposed to antibody before virus attachment to cells has decreasing amount of virus on cell surface with increasing concentrations of antibodies. This trend is also detected when the virus is exposed to antibodies after it has attached to cells, indicating that virus had been removed from the cell surface by antibodies. Notably, the HMAb 5J7 seemed to be more efficient in removing the virus, when mixed with virus after it had attached to cells than before attachment (Fig. 1d,e). Overall, the RT–PCR results suggest that the antibody binds either directly or very close to the receptor-binding site on the virus. In addition, HMAb 5J7–virus affinity is much higher than the virus–receptor affinity, thus displacing the virus on the cell surface.

In addition to inhibition of receptor binding by HMAb 5J7, the antibody can also prevent the virus entry into cells at a postattachment step. When 5J7 is first added to the virus and then incubated with cells (preattachment), the antibody reduces the number of bound virus particles but there is still a substantial fraction of virions (~50% at 10 ng μl^−1^) that bind to the cells (Fig. 1d). The preattachment neutralization test (Fig. 1c) showed that neut_50_ is about 0.56 ng μl^−1^ and neut_100_ at 10 ng μl^−1^. The fact that 10 ng μl^−1^ results in 100% neutralization (as shown in the neutralization test; Fig. 1c) despite substantial virus binding to cells (Fig. 1d) indicated that this MAb can block infection after virus attachment to cells.

### Mature DENV3 and DENV3–Fab 5J7 complex structures

**Figure 2:**
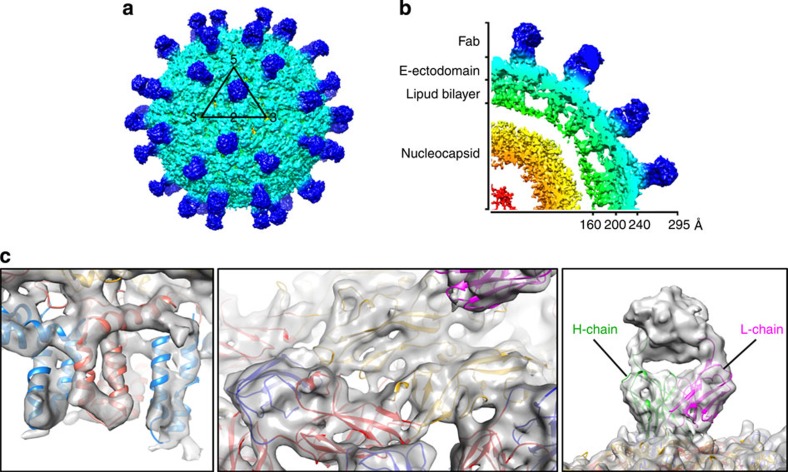
The cryo-EM structure of DENV3 complexed with Fab 5J7. (**a**) The 9-Å cryo-EM map of DENV3 complexed with Fab 5J7. The black triangle and numbers represent an icosahedral asymmetric unit and the icosahedral vertices, respectively. (**b**) Cross-section of a quarter of the cryo-EM map. Cryo-EM map is coloured by radii: red-to-yellow gradient (0–160 Å), green (160–200 Å), cyan (200–240 Å) and blue (240–295 Å). (**c**) The fit of transmembrane α-helices (left) and ectodomain (centre) of E proteins and the heavy and light variable region of antibody (right) into the density map. E protein DI, DII and DIII are coloured in red, yellow and blue, respectively. The stem and transmembrane region of the E protein is coloured in blue, whereas the same region in the M protein is coloured in salmon (left panel).

The cryo-EM structure of DENV3 complexed with Fab fragments of HMAb 5J7 was determined to 9 Å resolution (Fig. 2a,b). The shapes of the transmembrane helices and the domain boundaries of E protein and Fab molecules were well resolved (Fig. 2c). At full occupancy, 60 copies of Fab 5J7 bound to the virus surface.

The footprint of HMAb 5J7 on the E protein can be identified by fitting the crystal structure of the DENV3 E ectodomain (PDB accession code 1UZG)^13^ and the Fab homology model into the cryo-EM map. To increase the precision of the fit of E protein structures on DENV3 surface, we also determined the cryo-EM structure of uncomplexed mature DENV3 to 6 Å resolution (Supplementary Fig. 2a,b). The densities of the helical ridges on the transmembrane helices are visible (Supplementary Fig. 2d). DENV3 used in this structure determination was grown in C6/36 cells at 28 °C and kept at 4 °C before freezing on cryo-EM grids. As the DENV3-Fab 5J7 complex was formed at 37 °C, we also determined a 7-Å resolution cryo-EM structure of the uncomplexed mature DENV3 that had been exposed to 37 °C for 30 min and incubated at 4 °C for 2 h before freezing (Supplementary Fig. 3a,b). The DENV3 E protein has two residues deleted in the *E*_o_*F*_o_ loop (Supplementary Fig. 2e) compared with DENV1, 2 and 4. Superposition of cryo-EM structures of uncomplexed DENV3 at 28 °C and 37 °C showed similar structures (Supplementary Fig. 3c). Therefore, unlike the DENV2 New Guinea C strain^32^,^33^, the E protein layer of the uncomplexed DENV3 863DK strain does not undergo irreversible conformational changes when exposed to 37 °C (Supplementary Figs 3 and 4).

A homology model of the variable region of the Ab heavy and light chains (Fig. 2c), and the 6-Å resolution cryo-EM structure of the uncomplexed DENV3 863DK strain were then fitted into the 9-Å cryo-EM Fab-DENV structure. The DENV3–Fab 5J7 complex structure displayed a very similar E protein conformation as uncomplexed DENV3. Superposition of the Cα chains of E proteins of the Fab complex and uncomplexed cryo-EM structures showed a root mean square deviation value of 1.08 Å. This shows that binding of HMAb 5J7 did not induce significant structural rearrangement of the E proteins on virus surface.

### Epitope recognized by HMAb 5J7

**Figure 3:**
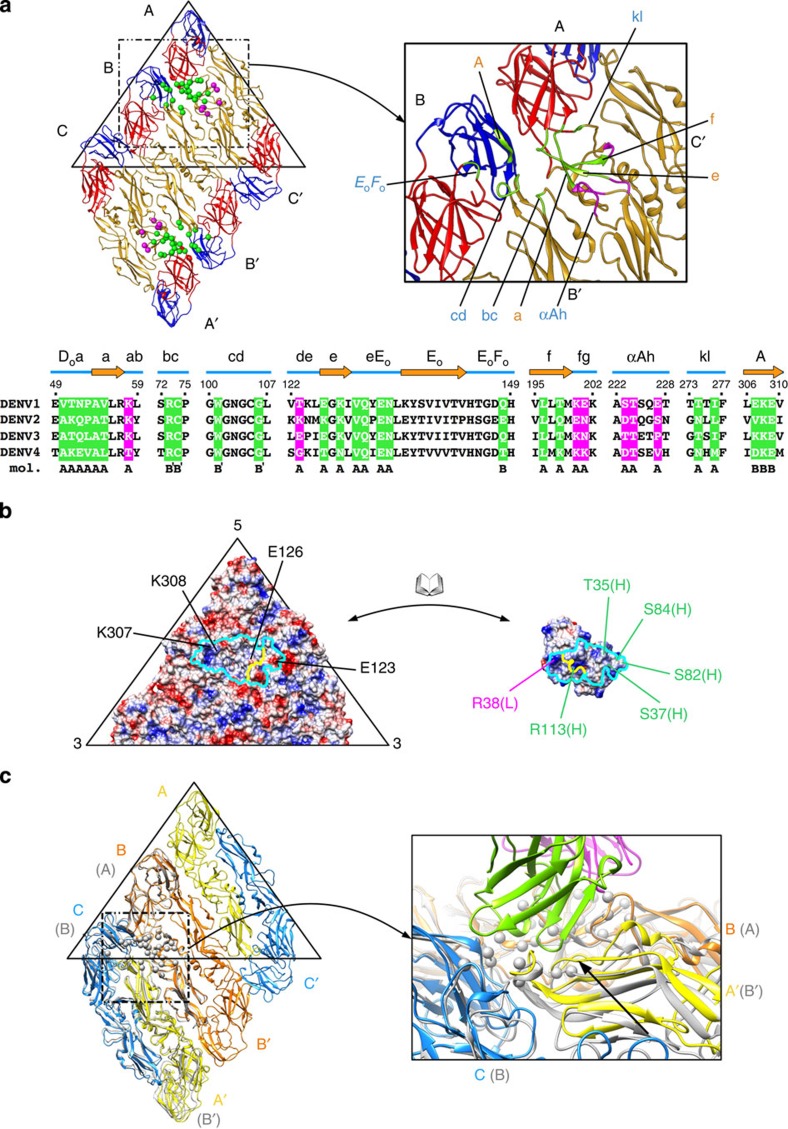
Fab 5J7 binds to three E proteins. (**a**) 5J7 epitope on an E protein raft. The three E protein molecules in an asymmetric unit are labelled as A, B and C, respectively, and those in the neighbouring asymmetric unit, A′, B′ and C′, respectively. The residues on the E protein that interact with heavy and light chains of the Fab are shown as green and magenta spheres, respectively. Bottom panel: comparison of epitope to the other DENV serotypes. The secondary structures are indicated above the amino acid sequence. The amino acid residues that interact with the heavy and light chain of Fab 5J7 are highlighted in the same colouring scheme as above. The individual E protein molecules in the two asymmetric units of the raft where the epitope residues belong are also indicated. (**b**) Open-book representation of the electrostatic potential of the interacting residues on the E proteins (left) and Fab 5J7 (right). Positive, negative and neutral charges are coloured in blue, red and white, respectively. Boundaries of the interacting interface on antibody and epitope are shown as cyan lines. The border between the heavy and light chains of the antibody paratope and its corresponding footprint on the epitope are coloured yellow. Interacting residues that show charge complementarity between the epitope and Ab are indicated. Residues on heavy and light chains are labelled with green and magenta colour, respectively. (**c**) Fab 5J7 does not bind to a similar epitope on the E protein B-C-A′ mols (left figure, dotted box) probably due to the difference in the conformation of the bc loop in DII. Superposition of the 5J7 epitope (spheres) on E protein mols A-B-B′ (grey), onto molecules B-C-A′ on the 6 Å resolution, uncomplexed DENV3 cryo-EM structure (left). Enlarged view (right) showed that the bc loop on mol B′ of the epitope on A-B-B′ (grey spheres) is shifted in mol A′ (black arrow) of the B-C-A′ molecules. The elevated position of the bc loop in mol A′ may lead to side-chain clashes with the heavy chain of the Fab molecule (green), thus preventing antibody binding.

As side-chain densities are not resolved in the 9 Å resolution cryo-EM Fab 5J7–DENV3 map, interacting residues between Fab and E proteins were identified by observing pairs of Cα atoms within a distance of <8 Å. In addition, the ability of these residues to form hydrogen bonds or hydrophobic interactions was also considered. One Fab molecule bound to three E proteins (molecules (mol) A, B and B′ of the E protein raft) (Fig. 3a). The footprint of the antibody covers a surface area of ~1,500 Å^2^ of which ~74% (~1,100 Å^2^) is on mo) A (Fig. 3a). The large area of contact on a single E protein molecule is consistent with the ability of HMAb 5J7 to bind to rE protein^26^ at high antibody concentrations (Supplementary Fig. 1).

The interacting surface on the Fab 5J7 molecule comprises five of the six common complementary determining regions: L1 (residues 35–38), L3 (residues 99–101), H1 (residues 35–38), H2 (residues 59–62) and H3 (residues 105–113) with the addition of H2–3 region (residues 81–84) (Table 1). In most antibodies, the H2–3 region is located on the outer part of complementary determining regions and usually does not interact with the antigen. However, in Fab 5J7, the H2–3 region interacted with DIII of E protein mol B. A total of 15 amino acid residues from the variable region of the heavy chain interacted with E proteins. In comparison, only six amino acids from the L chain are involved.

The 5J7 epitope probably consists of 31 amino acid residues (Fig. 3a). Interactions between Fab 5J7 and E protein on DENV3 consist of hydrogen bonds, salt bridges and hydrophobic interactions. For example, R38 (light chain) and R113 (heavy chain) of Fab 5J7 interact with E123 and E126 of E protein, respectively. A slightly negatively charged patch formed by T35, S37, S82 and S84 of the heavy chain interacted with a positively charged patch formed by K307 and K308 of the E protein (Fig. 3b). Fab 5J7 binds to the DI–DII hinge region of mol A, DIII of mol B and the tip of DII of mol B′, which contains the fusion loop (Fig. 3a and Table 1). The three E protein domains comprising the epitope are each crucial for discrete components of the mechanisms underlying DENV attachment and entry. DIII is involved in receptor binding and fusion^34^,^35^. DI–DII hinge is important for movements of the E protein during virus entry. The DII fusion loop is essential for binding to the endosomal membrane during fusion^17^. Previous study^26^ also identified parts of the 5J7 epitope by sequencing neutralization escape mutant. The escape mutant contains a lysine insertion in the DI–DII hinge region between residues Q269 and N270, consistent with the epitope identified by cryo-EM.

Another site on the E protein raft that is similar to the quaternary structure-dependent epitope recognized by HMAb 5J7 is located on mols B, C and A′ (Fig. 3c). However, antibody binding was not detected in this area. Fabs bound to the epitope on mols A, B and B′ would not hinder the binding of Fabs to this second site, as there is enough space between the Fabs on those two sites (Supplementary Fig. 5). Superposition of the Fab-E protein structure onto this site in the 6-Å resolution uncomplexed DENV3 structure (Supplementary Fig. 3) showed that the bc loop of mol A′ is elevated compared with that in mol B′ (Fig. 3c). The elevated position resulted in a distance of ~3 Å between Cα chains of the bc loop of mol A′ and the heavy chain of the Fab molecule. This would probably result in side-chain clashes, thus preventing antibody binding.

## Discussion

**Figure 4:**
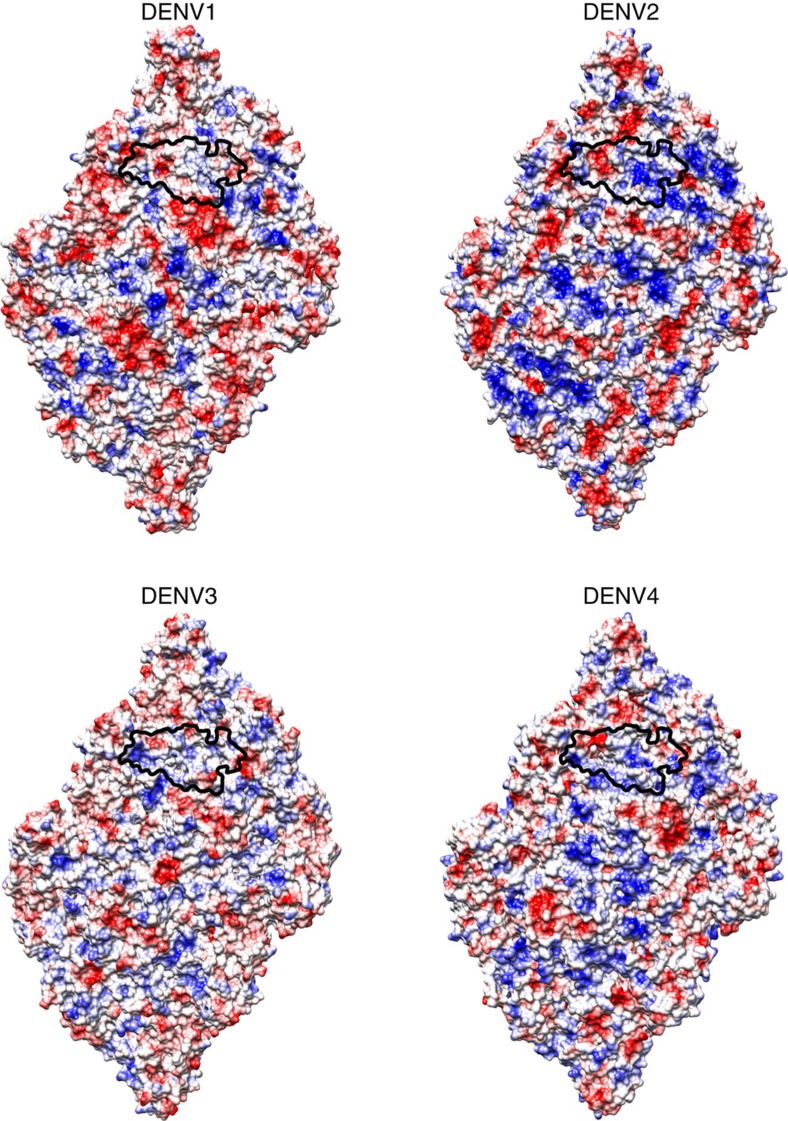
Comparison of the electrostatic charges of the epitope across DENV1–4. Electrostatic potential surfaces of a raft consisting of two icosahedral asymmetric units are shown. Positive and negative charges are shown in blue and red colours, respectively. The border of the 5J7 epitope is outlined with a black line. The DENV structures used for the electrostatic potential surface calculations are from PDB accession codes 4CCT (DENV1), 3J27 (DENV2), 3J6S (DENV3) and 4CBF (DENV4).

HMAb 5J7 binds to DENV3 specifically. Comparison of the DENV3 amino acid sequence (Fig. 3a) and surface electrostatic potential (Fig. 4) of the regions corresponding to the epitope with the other serotypes showed that some residues on DENV1, 2 and 4 have opposite charges. For example, in DENV3, positions 123 and 126 contained negatively charged glutamate residues that interact with a positive patch on Fab 5J7, whereas the same positions in DENV2 are occupied by positively charged lysine residues. Other residues corresponding to the 5J7 epitope in DENV1, 2 and 4 also have different hydrophobicity characteristics compared with DENV3. For instance, position 123 of DENV4 is occupied by glycine, thus losing the ability to form a salt bridge with R38 of the Fab molecule. In DENV3, position 55 (Thr) and 307 (Lys) formed hydrogen bonds with residues K107 and S82/S84 of the Fab molecule, respectively. On the other hand, DENV1 and DENV4 at position 55 and also in DENV2 at position 307 contained hydrophobic residues. In addition, DENV1, 2 and 4 E proteins have two additional amino acids between residues 156 and 157 (*E*_o_*F*_o_ loop) (Supplementary Fig. 2e). As the Fab heavy chain binds to a nearby residue (Q148) in this loop, the differences in the length of *E*_o_*F*_o_ loop and the amino acid sequence variation of the epitope may determine the serotype specificity of HMAb 5J7

**Figure 5:**
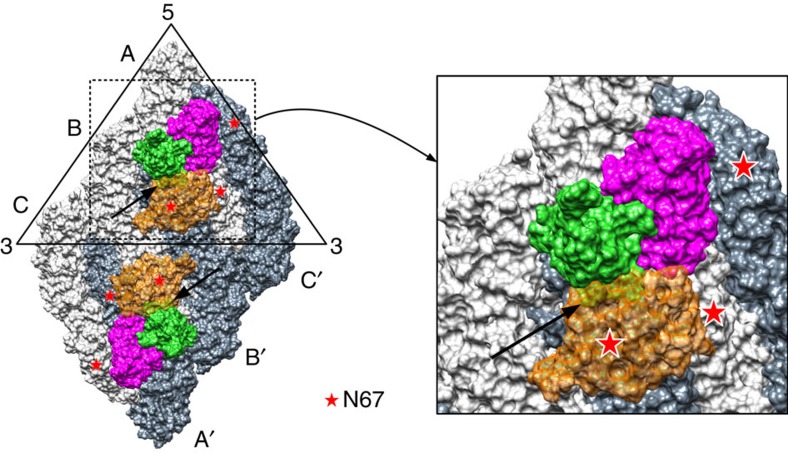
Simultaneous binding of Fab 5J7 and DC-SIGN to DENV3 surface is unlikely to occur. Superposition of the carbohydrate recognition domain (CRD) of DC-SIGN–DENV2 structure onto Fab 5J7–DENV3 structure showed the Fab 5J7 and DC-SIGN molecules clashed on the virus surface. One DC-SIGN molecule binds across two adjacent N67 glycans on the virus surface. Simultaneous binding of these molecules on the virus surface is not possible, as the Fab and DC-SIGN molecules clashed. E protein mols A, B and C are coloured in light grey and mols A′, B′ and C′ in grey. The heavy and light chains of the Fab are coloured in green and magenta, respectively. DC-SIGN is shown as a transparent orange surface. The glycosylation sites on N67 are marked as red stars. Arrows indicate the clashes between the Fab and DC-SIGN molecules.

RT–PCR quantification of virus that were exposed to HMAb 5J7 after virus binding to cells showed that the antibody is able to remove virus from cell surface, therefore suggesting that the antibody is binding directly or close to the receptor binding site. Interestingly, comparing the amounts of virus on the cell surface that are exposed to antibodies pre- and post attachment suggests that the HMAb 5J7 is more efficient in stripping the virus off the cell surface when it has already bound to its receptor (Fig. 1e). As HMAb 5J7 binds across three E proteins, any motions between these molecules may reduce antibody binding. Our uncomplexed DENV3 structure represents the structure that was incubated at 37 °C for 30 mins and then 4 °C for 2 h before freezing. It did not show any structural difference compared with the sample that was grown at 28 °C. This finding suggests that the E proteins on DENV3 do not undergo temperature-irreversible conformational change, as observed in DENV2 (refs 32, 33). However, it is still possible that the E proteins have small conformational changes when kept at 37 °C, thus lowering the binding of HMAb 5J7 to virus before it attaches to cells. When the virus attaches to a receptor, this engagement may stablize the virus quaternary structure, thus improving the binding efficiency of the antibody consistent with our RT–PCR results. The DENV receptor(s) on Vero cells, which were used in the RT–PCR pre- and postattachment assays, are largely unknown. One receptor that has been identified previously is the carbohydrate recognition domainsof the dendritic cell-specific intercellular adhesion molecule 3-grabbing non-integrin (DC-SIGN). One molecule of DC-SIGN binds across two N67 glycosylation sites from neighbouring E proteins (mol A and B′) (Fig. 5)^36^. Superposition of the DENV–carbohydrate recognition domain and DENV3–Fab 5J7 complex structures showed a clash between DC-SIGN and the heavy chain of Fab 5J7, suggesting that the binding of antibody may prevent or compete with the virus from interacting with the DC-SIGN receptor (Fig. 5) on dendritic cells.

HMAb 5J7 can also block infection at a step post virus attachment, probably fusion of virus to the endosomal membrane. The two Fab molecules on an E protein raft can lock four E proteins (mols A, B, A′ and B′) into a rigid configuration (Fig. 3a). E proteins on the uncomplexed DENV undergoes structural change when exposed to low pH environment in the endosome during fusion^17^. This involves the reorganization of the E proteins from a dimeric structure to a trimeric one^17^,^37^. In the Fab 5J7-DENV structure, only the E protein mols C remain unbound by Fabs, suggesting that the switch to trimeric structure may not be possible.

**Figure 6:**
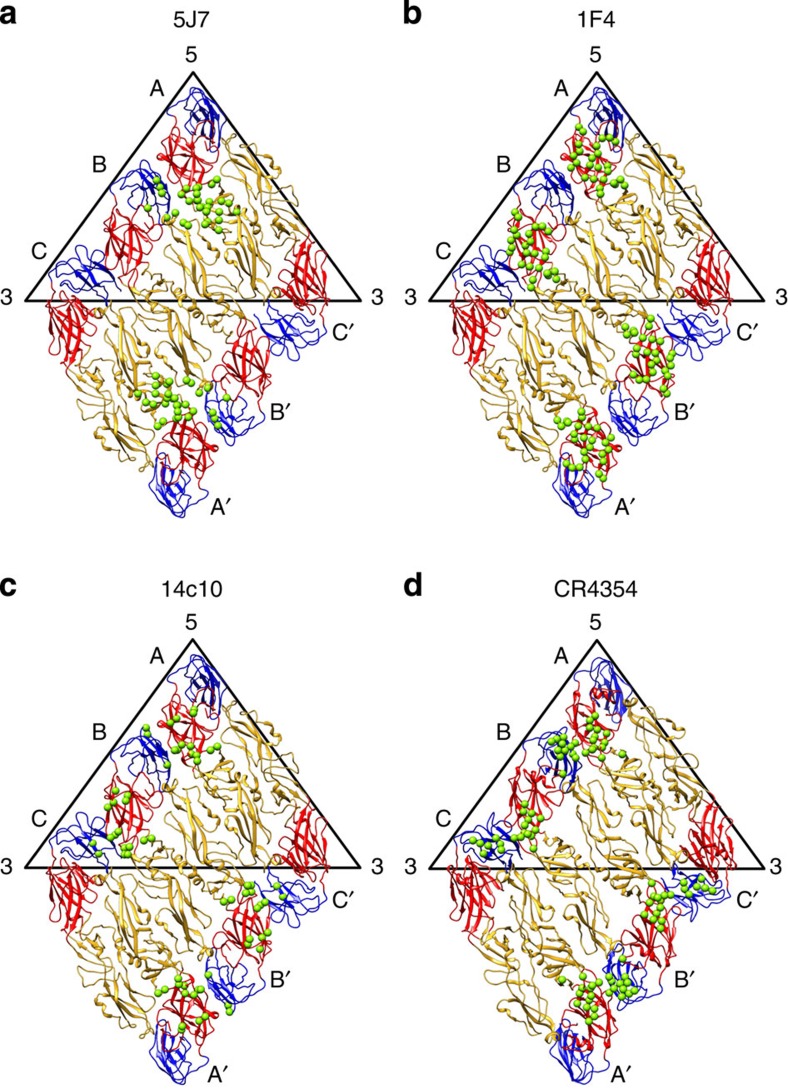
Comparison of quaternary structure-dependent epitopes on E protein raft. Comparison of quaternary structure-dependent epitopes on E protein raft bound by HMAbs (**a**) 5J7, (**b**) 1F4 (ref. 38) and (**c**) 14c10 (ref. 29) to DENV, and (**d**) HMAb CR4354 to West Nile virus (WNV)^39^. Epitopes are represented as green spheres.

Recognition of quaternary structure-dependent epitope is common among some strongly neutralizing HMAbs^26^ and this could be due to peculiar arrangement of the E proteins lying flat to each other on the virus surface. The binding of antibodies across the E proteins will then prevent structural rearrangment necessary for infection such as fusion. Structures of HMAbs that bind the E DI–II region of DENV1 (1F4 and 14c10)^29^,^38^ and West Nile virus (CR4354)^39^ had been determined previously (Fig. 6). These antibodies are serotype specific and bind to quaternary structure-dependent epitopes. HMAbs 14c10 and CR4354 bind to overlapping epitopes on DENV1 and West Nile virus, respectively (Fig. 6c,d). The epitopes are located on DI and DI–II hinge region of an E protein and DIII of an adjacent E protein. Although HMAb 1F4 binds to the DENV1 particle but not rE protein, the epitope is located on a E protein monomer at the DI–II hinge (Fig. 6b). Binding of HMAb 1F4 is highly sensitive to the DI–DII hinge angle. This is consistent with the preference of the Ab to bind to the E DI–DII hinge on the virus surface but not to the same region on soluble rE protein, as the hinge angle of the DI–DII region is highly variable in soluble rE protein. These HMAb-flavivirus cryo-EM structures showed that 120 molecules of Fab bind to virus surface at full occupancy. HMAb 5J7, on the other hand, binds to three E proteins at the same time, therefore requiring only 60 copies for full occupancy, consistent with its higher neutralization potency. This finding of a single Fab simultaneously engaging three functionally significant E protein domains each on a different E protein molecule has not been observed in antibodies binding to other viruses. In conclusion, the very low quantity of Ab required for neutralization and the unique mechanism of binding and neutralization mediated by interaction with three critical E protein domains make HMAb 5J7 a potential therapeutic candidate molecule. The epitope also posed as an interesting target for structure-based rational vaccine design.

## Methods

### Virus sample preparation

Primary isolate DENV-3/SG/05K863DK1/2005 (ref. 40) and the reference strain DENV3 CH53489 (genotype *2*, a Bangkok isolate that was isolated by USAMC-AFRIMS in 1973) were used in this study. DENV3 CH53489 was used in all the experiments except for the cryo-EM studies, where the primary isolate was used. Neutralization test using HMAb 5J7 showed that the antibody strongly neutralized both strains.

The methods to produce and purify virus had been described previously^9^. Briefly, DENV3 were grown in *A. albopictus* C6/36 cells. Cells were grown to ~80% confluency before inoculating with DENV at a multiplicity of infection of 0.1. Inoculated cells were incubated at 28 °C for 4 days. The virus-containing supernatant was harvested and clarified by centrifugation at 9,000*g* for 30 min. Virus in supernatant was precipitated by using 8% w/v polyethylene glycol 8000 in NTE buffer (10 mM Tris-HCl pH 8.0, 120 mM NaCl and 1 mM EDTA). The pellet was collected by centrifugation at 14,300*g*. Virus was resuspended in NTE buffer and then further purified by centrifugation using a 24% w/v sucrose cushion and then a linear 10% to 30% w/v potassium tartrate gradient. The virus band was collected, buffer exchanged into NTE buffer and concentrated using an Amicon Ultra-4 centrifugal concentrator (Millipore) with a 100-kDa molecular-mass cut-off filter. All steps of the purification procedure were done at 4 °C. Purified virus was kept at 4 °C before freezing on cryo-EM grids. Coomasie-stained SDS–PAGE gel was used to assess the purity of the virus preparation. A faint band of the 25-kDa premembrane protein was detected, indicating a slight contamination with immature virus particles. Concentration of the envelope protein was estimated by comparing the band with the different concentrations of BSA protein standard band.

### DENV3 neutralization assays

The flow cytometry-based neutralization assay with Vero-81 cells were conducted using a similar protocol similar to the neutralization assay with U937+DC-SIGN cells described in Krauss *et al.*^41^ Briefly, 1.25 × 10^4^ cells per well were plated in a 96-well plate 48 h before conducting the experiment. The 5J7 whole antibody (mAb) or 5J7 Fab were serially diluted and incubated with DENV3 (CH53489) for 1 h at 37 °C under 5% CO_2_. The mixture was then added to the 96-well plate containing Vero-81 cells. Two hours post infection, the cells were washed twice with fresh infection media and incubated at 37 °C under 5% CO_2_. At 24 h post infection, the cells were washed, fixed, permeabilized and stained for DENV E protein using 2H2 conjugated to Alexa Fluor 488, and percentage of infected cells were measured using flow cytometry. Sigmoidal neutralization curves were generated using GraphPad Prism 6.

### ELISA assay to determine the serotype-specificity of HMAb 5J7

Fifty microlitres of polyethylene glycol-precipitated virus was diluted in PBS and used to coat Maxisorp ELISA plates (Nunc) overnight at 4 °C. The wells were blocked using 300 μl of 5% (w/v) skimmed milk-PBS per well for 1 h. Fifty mirolitres of HMAb 5J7 at 5 μg ml^−1^ was applied to each well for 1 h at 37 °C and the plates washed thrice with PBS-Tween (0.1% v/v). This was followed by the addition of 50 μl of goat anti-human IgG-horseradish peroxidise-conjugated antibody (Invitrogen, 1:2,000 v/v) for another hour at 37 °C. Following a second wash with PBS-Tween, detection was achieved using TMB chromogen substrate (Life Technologies) and the reaction stopped with 50 μl of 1 M HCl. Absorbance was read at OD 450 nm.

### ELISA assay to compare 5J7 binding to rE protein and virus particle

The antigens used for the ELISA assay were from DENV3 strain CH53489. Intact virions or rE protein (amino acids 1–395) were purified as previously described^23^,^42^. HMAbs used in the assay were 1M7 and 5J7. 1M7 is a flavivirus cross-reactive antibody that binds to a conserved epitope on the fusion loop of the rE protein^24^. Mouse MAbs used in the assay were 4G2 and DV2-30. 4G2 is a flavivirus cross-reactive antibody that binds to a conserved epitope on the fusion loop of the rE protein^43^. DV2-30 is a DENV2 type-specific antibody that was used as a negative control^44^. ELISA plates (Nunc 242757) were coated overnight at 4 °C with 50 ng of virus or 100 ng E protein in carbonate buffer. Plates were washed and blocked with 3% normal goat serum in PBS (blocking buffer). Antibodies were serially diluted in blocking buffer and added to each well for 1 h at 37 °C. The plates were washed and then incubated with alkaline phosphatase-conjugated goat anti-mouse or human IgG secondary antibodies for 1 h at 37 °C. After a final wash step, the plates were developed using p-nitrophenyl phosphate substrate and any colour change was quantified with a spectrophotometer.

### Pre- and postattachment neutralization assays

The pre- and postattachment neutralization assays were conducted as previously described^38^. Briefly, for preattachment neutralization assays, Vero-81 cells were grown in 24-well plates and all reagents were cooled for at least 30 min at 4 °C. Serially diluted 5J7 antibody was combined with DENV3 virus and incubated at 4 °C for 1 h. The mixture was added to Vero cells and then incubated for 1 h at 4 °C. One hour post infection, the infected cells were washed three times with cold PBS, methyl cellulose was then added and kept at 37 °C for 4 days before fixing and staining the cells for DENV foci. For postattachment neutralization assays, Vero-81 cells and other reagents were prechilled at 4 °C. DENV3 virus was added to Vero cells and incubated for 1 h at 4 °C. The virus-bound cells were then washed twice with cold PBS, serially diluted 5J7 antibody was added to cells and further incubated for 1 h at 4 °C. The cells were washed once with cold PBS and incubated for another 4 days at 37 °C. The cells were then fixed and stained for DENV foci.

### RT–PCR to quantitate virus on cell surface

The amount of DENV3 remaining on the surface of Vero cells after 5J7 treatment was estimated by quantitative RT–PCR as previously described^45^. In brief, DENV3 was mixed with different concentrations of 5J7 before and after the virus attached to cells at 4 °C. The cells were washed and total cellular RNA was purified using TRIzol reagent (Lifesciences), as described in the manufacturer’s instructions. Complementary DNA was prepared using iScriptTM Reverse transcription supermix for RT–qPCR kit (Bio-Rad), as described in the manufacturer’s instructions. The 20-μl reaction contained 4 μl iScript RT supermix (5 × ), RNA template (1 μg) and nuclease-free water. The RT–PCR condition to synthesize the first strand of cDNA are: 25 °C for 5 min for attachment of primers to template RNA), 42 °C for 30 min for reverse transcription and final 85 °C for 5 min for reverse transcription inactivation.

Real-time quantitative PCR (qPCR) was performed using iQTM SYBRR Green supermix kit (Bio-Rad) in a CFX 96 Real-Time System (Biorad), as per the manufacturer’s instruction. The 20-μl, reaction contained 10 μl SYBR Green super mix (2 × ), 1.2 μl each of 10 mM forward (5′-CAA TAT GCT GAA ACG CGA GAG AAA-3′) and reverse primers (5′-AAG ACG TAA ATA GCC CCC GAC-3′), 3 μl cDNA and 4.6 μl double-distilled H_2_O. The thermal profile for qPCR was 95 °C for 3 min, to activate the polymerase and denature DNA; this is then followed by 40 cycles of denaturation at 95 °C for 10 s, annealing at 65 °C for 10 s, extension at 65 °C for 30 s. Glyceraldehyde-3-phosphate dehydrogenase was used as the housekeeping gene to normalize samples (forward 5′-CTGTTGCTGTAGCCAAATTCGT-3′, reverse 5′-ACCCACTCCTCCACCTTTGAC-3′). The analysis of relative levels of DENV3 RNA in different samples was performed by comparative 2-ΔΔ*C*T method^46^.

### Cryo-EM image collection and processing

Purified virus was mixed with Fab 5J7 in the same molar concentration as the E protein in DENV3 and incubated at 37 °C for 30 min followed by another 2 h at 4 °C. The DENV3 control (without Fab 5J7) was also prepared in similar way, except it was incubated at two different temperatures (4 °C and 37 °C) for 30 min. From this point onwards, in this study, these samples are designated as DENV3 at 28 °C (the temperature at which it was grown) and DENV3 at 37 °C, respectively. A 2.5-μl sample was transferred onto an ultra-thin carbon-coated lacey carbon grid (Ted Pella) and blotted with filter paper for 2 s before snap freezing in liquid ethane, using the FEI Vitrobot Mark IV. Frozen grids were stored in liquid nitrogen temperature. The virus particles were imaged using Titan Krios cryo-EM with field-emission gun operated at 300 kV. Images were collected at nominal magnifications of 59 k (DENV3—Fab 5J7 complex), 75 k (DENV3 at 28 °C control) and 47 k (DENV3 at 37 °C), with a total electron dose of 18–20 e Å^−2^. The images were captured on a direct electron detector (Falcon, FEI) for DENV3-Fab 5J7 and DENV3 at 37 °C data sets, and a Gatan charge-coupled device camera for DENV3 at 28 °C data set with effective pixel sizes of 1.37, 1.7 and 1.16 Å per pixel, respectively. Images showing strong drift and astigmatism were discarded. In total, 254 (DENV3—Fab 5J7 complex), 836 (DENV3 at 28 °C) and 138 images (DENV3 at 37 °C) were selected with a defocus range of 0.4–4.2, 1.1–3.5 and 0.8–3.2 μm, respectively, for further processing.

### Cryo-EM image reconstruction

The spiky-looking unbroken particles of the DENV3-Fab 5J7 complex were selected manually, by using the e2boxer tool in the EMAN2 (ref. 47) software package. In the control DENV3 at 28 °C and 37 °C, smooth particles that represent the majority of the virus population were selected. In total, 4,144 (DENV3–Fab 5J7 complex), 11,700 (DENV3 at 28 °C) and 4,035 (DENV3 at 37 °C) particles were used for reconstruction. The parameters of the contrast transfer function for each micrograph were first automatically determined by using the programme *fitctf* and then manually optimized using the *ctfit* programme in EMAN^48^.

For the DENV3 at 28 °C control, the cryo-EM map of DENV4 (Electron Microscopy Database accession code EMD-2485) was used as an initial model. The initial model was used in the orientation search of the particles that was carried out using Multi-Path Simulated Annealing procedure^49^. Following the orientation search, a three-dimensional reconstruction was done with make3d from EMAN. A total of 6,800 particles was used in the reconstruction of the final map of DENV3 at 28 °C.

For the DENV3 at 37 °C control and also the Fab 5J7 complex, the cryo-EM map of DENV3 at 28 °C was used as an initial model. The final map of DENV3 at 37 °C was reconstructed from 3,009 particles. For the reconstruction of the DENV3–Fab 5J7 complex, the particles were initially treated as a homogenous population but a medium resolution map (~15 Å) was obtained. The micrograph of DENV3 at 28 °C or 37 °C controls showed that there are ~20% of immature particles. To reduce the influence of immature and unbound mature virus particles in the Fab 5J7–DENV3 reconstruction, several cycles of reconstruction using simultaneously three initial models—DENV3–Fab 5J7, DENV3 at 28 °C and immature DENV (Electron Microscopy Database accession number EMD-2141)) were done. From 4,144 particles, 2,047 particles (49%) were selected for three-dimensional reconstruction of the DENV3–5J7 complex, whereas 863 (20.8%) and 731 (17.6%) particles were selected for mature and immature virus reconstruction, respectively. This procedure allowed the identification of the likely unbound mature and immature particles, and these particles were removed in subsequent cycles of the DENV3–Fab 5J7 reconstruction. The final map of the DENV3–Fab 5J7 complex was reconstructed from 970 particles. The resolution of the final map was estimated by observing 0.5 cutoff value of the plot of the Fourier shell correlation coefficient between two reconstructed maps each built from half of the data set. The final resolutions of the DENV3–Fab 5J7 complex, DENV at 28 °C and at 37 °C were 9, 6 and 7 Å, respectively.

### Model fitting

Model fitting of DENV3 control at 28 °C was done first, because the resolution of the map was higher (6 Å) than the DENV3 control at 37 °C map (7 Å). This approach allowed for precise fitting. Although structure of recombinant DENV3 E-protein ectodomain is known, we did not use it for initial fitting as the structure does not contain the stem and transmembrane regions. Furthermore, the DI–DII hinge angle is different between the virus structure and crystal structures. Because of this, the cryo-EM structure of DENV2 (PDB code: 3J27) was initially used to fit the map and the residues were later mutated to that of DENV3 strain 863DK. Initial fitting was done manually by using the programme Chimera^50^ and the fit was further optimized using the ‘Fit in Map’ function in Chimera. The amino acid residues of the fitted DENV2 E and membrane proteins were then mutated to DENV3 sequence and the *E*_o_*F*_o_ loop was replaced by the crystal structure of DENV3 E protein, as it has a shorter loop than DENV2. Both procedures were done using COOT^51^. The molecular dynamic flexible fitting^52^ was used together with NAMD^53^ and VMD^54^ for cryo-EM map-guided molecular dynamic simulation to optimize the fit of the molecules. Symmetry restraints were applied to avoid clashes between neighbouring molecules and a factor of 0.5 was used to weigh the contribution of the cryo-EM map in the overall potential energy of the molecular dynamic simulation. The simulation involved 20,000 steps of minimization followed by 100,000 steps of molecular dynamics before converging into a stable solution. The final structures were observed to be free of misfits and clashes by using the O programme^55^.

The DENV3 control at 28 °C structure was then used to fit into the DENV3 control at 37 °C (7 Å resolution) and also the Fab 5J7 complex (9 Å resolution) cryo-EM maps. No obvious structural differences of the E proteins between these cryo-EM structures were detected. For the fitting of the Fab 5J7 molecules into the antibody-complexed cryo-EM map, a homology model for Fab 5J7 was built by using Swiss-model server^56^ with two human Ab structures (PDB codes 3MA9 and 1DEE) as templates for the Fab 5J7 heavy and light chains, respectively. Only the variable region of the Fab structure was modelled. The variable regions of the Fab 5J7 heavy and light chains showed a high sequence homology of 78% and 90%, respectively, with the corresponding variable regions in the aforementioned PDB starting homology models (Supplementary Fig. 6). Fitting procedure was done in a similar manner as the DENV3 control.

### Structural analysis

We carried out structure and sequence analyses using the following computer programmes: Gerstein’s accessible surface calculator^57^ in StrucTools server ( http://helixweb.nih.gov/structbio/basic.html) for calculating the contact area between two molecules, Multalin^58^ server for multiple sequence alignment and LSQ Superpose in COOT for superimposition of protein structures. Chimera was used for electrostatic potential surface calculation and visualization of the structures. The amino acid sequences used for comparison were DENV1 strain PVP159 (GenBank accession code AEM92304.1), DENV2 strain New Guinea—C (GenBank accession code AAA42941.1), DENV3 strain 863DK (GenBank accession code EU081190.1) and DENV4 strain Dominic 1981 (GenBank accession code AAK01233.1). The DENV structures used for electrostatic potential surface analyses were the cryo-EM structures of DENV1, DENV2 and DENV4 (PDB accession codes 4CCT, 3J27 and 4CBF, respectively).

## Author contributions

S.-M.L. and J.E.C. supervised the project. G.F., J.L.T. and V.A.K. did the cryo-EM image reconstruction. G.F. interpreted the cryo-EM maps and conducted the structural analysis. J.L.T. conducted neutralization and ELISA binding assays. P.K. purified the virus for cryo-EM studies. S.A.S. and J.E.C. prepared the Ab and Fab. T.-S.N. froze the sample and collected cryo-EM data. R.d.A., R.S.J. and A.d.S. conducted neutralization and attachment assays. G.F., J.L.T., R.d.A., A.d.S., J.E.C. and S.-M.L. wrote the manuscript.

## Additional information

**Accession codes:** The cryo-EM maps of the DENV3–Fab 5J7 complex, DENV3 at 28 °C and at 37 °C were deposited in the Electron Microscopy Database under accession codes EMD-5935, EMD-5933 and EMD-5934, respectively. The modelled DENV3 E protein–Fab 5J7 complex, DENV3 at 28 °C and at 37 °C structures were deposited in the Protein Data Bank under accession codes 3J6U, 3J6S and 3J6T, respectively.

**How to cite this article:** Fibriansah, G. *et al.* A highly potent human antibody neutralizes dengue virus serotype 3 by binding across three surface proteins. *Nat. Commun.* 6:6341 doi: 10.1038/ncomms7341 (2015).

## Supplementary Material

Supplementary Information Supplementary Figures 1-6.

## Figures and Tables

**Table 1:** Interacting residues between the Fab 5J7 and the E proteins on a raft.

**HMAb 5J7**	**E protein mols**			**HMAb 5J7**	**E protein mols**		
**H-chain**	**A**	**B**	**B**′	**L-chain**	**A**	**B**	**B**′
T35	Q52Q131E133N134	K308E309		S35	E123		
S37		K308	G106	S37	E123K200		
S38	Q52Q131N134			R38	E123K200N201		
I59	A54			Q99	K58T223		
V61	A54		C74	Y100	K58P227		
F62	A54		R73C74	I101	T223T224		
K81			W101				
S82		Q148K307					
S84		K307					
R105	Q52						
K107	L53T55						
L109	T51T274						
L110	A50L53K128V130L196T274I276						
F111	T198T274						
R113	E126						
