# Supplementary material for: A highly potent human antibody neutralizes dengue virus serotype 3 by binding across three surface proteins
Source: Nat Commun. 2015 Feb 20;6:6341. doi: 10.1038/ncomms7341 (PMC4346626; doi:10.1038/ncomms7341)
Supplement: Supplementary Information — Supplementary Figures 1-6. [file ncomms7341-s1.pdf]

Supplementary Figures

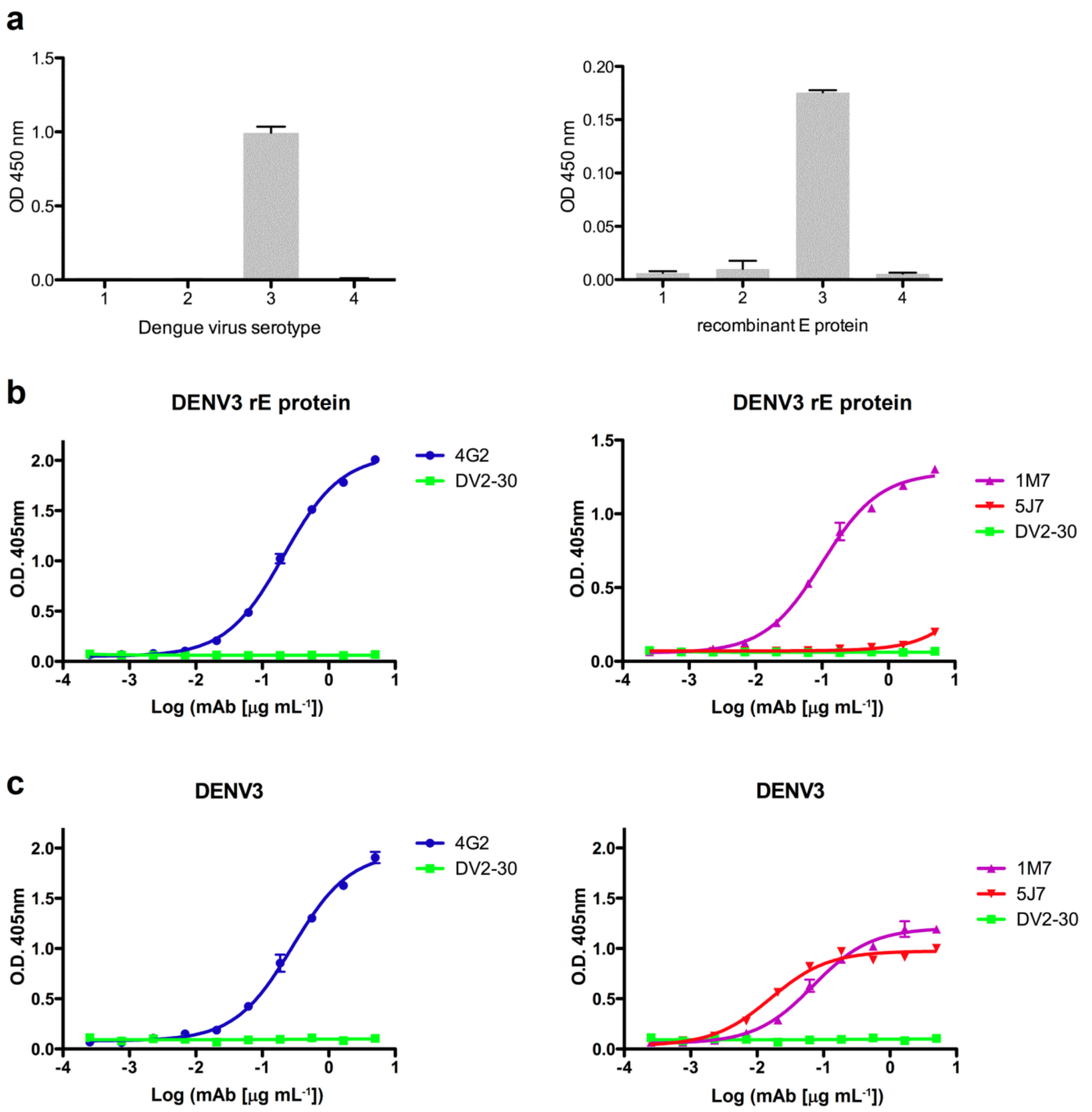

Supplementary Figure 1. HMab 5J7 binds specifically to DENV3 serotype and has higher affinity to whole virus than rE protein. (**a**) HMAb 5J7 showed specific binding to DENV3 particles (left) and rE protein (right) coated on ELISA plates. HMAb 5J7 binds to (**b**) rE protein at much lower affinity compared to (**c**) whole virus. Mouse MAb 4G2 (left panels in (b) and (c)), which bind to an epitope that is not quaternary structure dependent, is used as a control to show the

amount of rE and virus coated on the ELISA plates are similar. HMAb 1M7 (right panel in (b) and (c)), binds to rE and whole virus equally well. In comparison, HMAb 5J7 binds to whole virus with much higher affinity than rE protein suggesting that the preservation of quaternary structure of the virus is important for antibody binding. DV2-30 binds to DENV2 specificity and is used as a negative control. Values are mean ± standard deviation. Experiments were repeated at least twice.

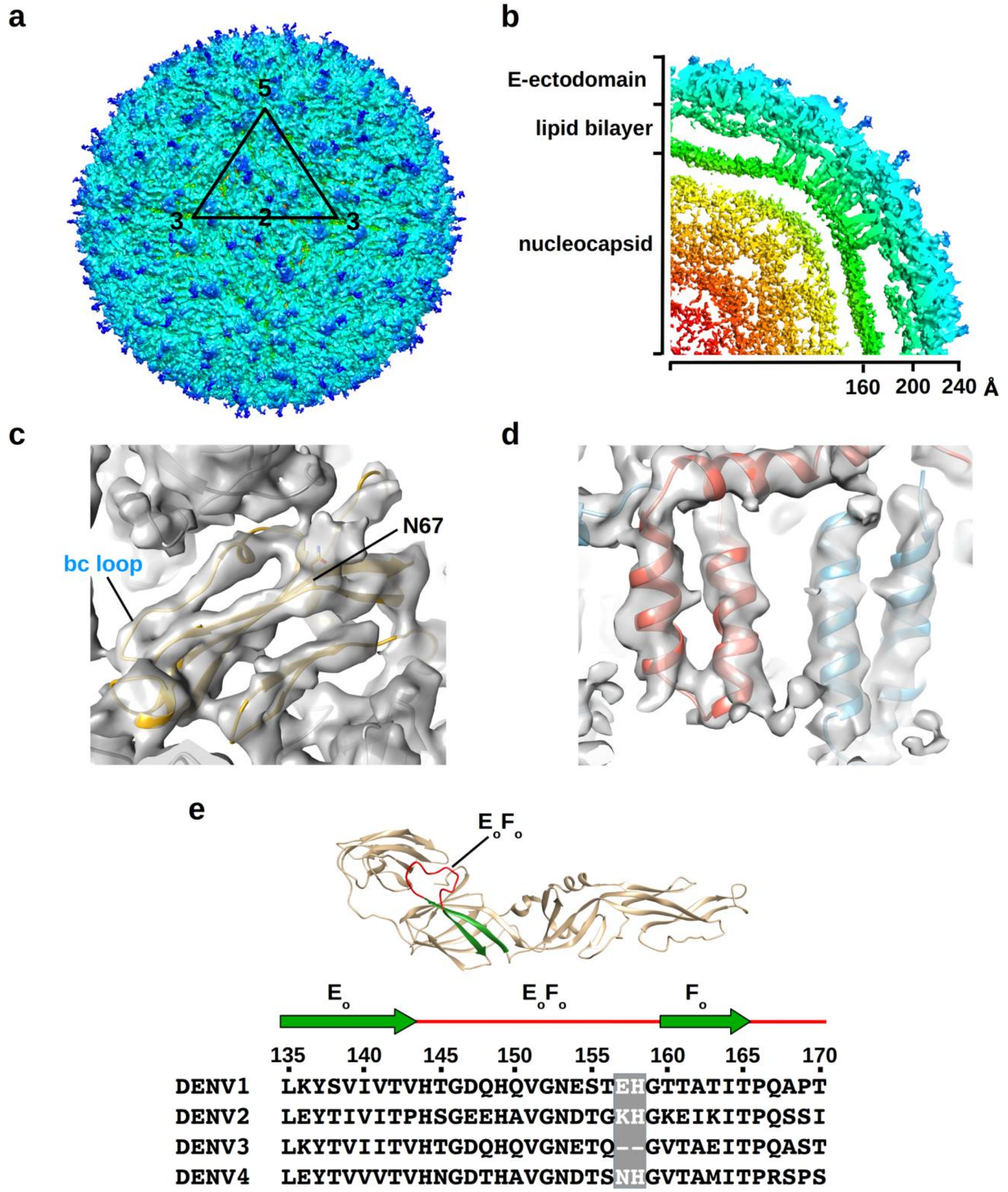

Supplementary Figure 2. The cryo-EM structure of mature DENV3 incubated at 28°C.

(**a**) The 6Å resolution cryo-EM map of mature DENV3. The E protein layer is colored in cyan and the glycosylation sites are colored in blue. Black triangle indicates an icosahedral asymmetric unit and the numbers represents icosahedral vertices. (**b**) Cross-section of a quarter of the cryo-EM map. Zoomed-in view of fitted (**c**) E ectodomain, and the (**d**) trans-membrane α-helices in the cryo-EM

densities (gray). DI, DII and DIII of the E ectodomain, membrane helices of the E protein and M-protein are colored in red, yellow, blue, light blue and salmon, respectively. Black arrow in panel (**c**) points to the density corresponding to glycosylation site at N67. (**e**) DENV3 has two amino acids deletion on the E_0F_0 loop when compared to other serotypes. The position of amino acids deletion in the sequence is shown in grey box.

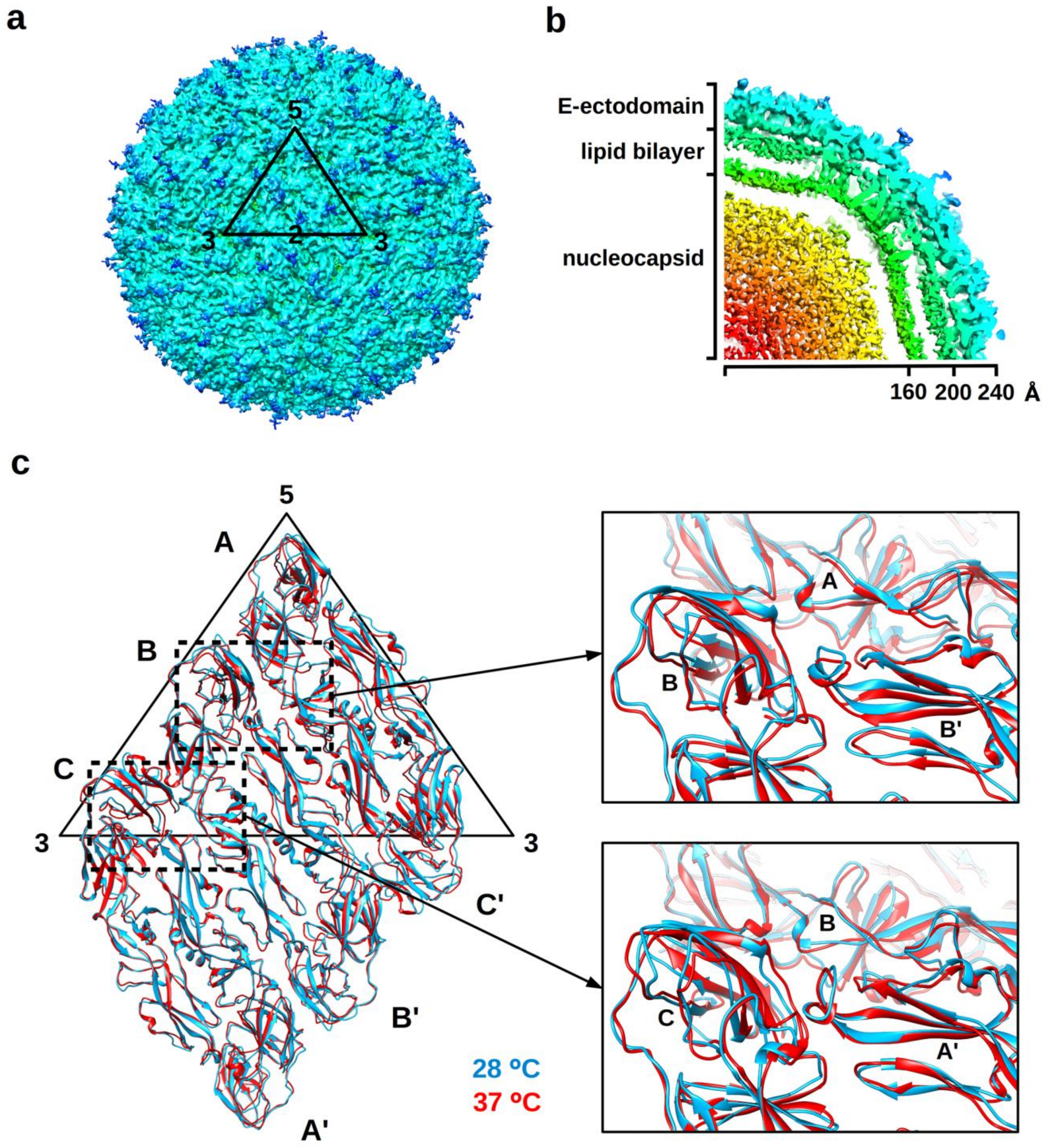

Supplementary Figure 3 The cryo-EM structure of mature DENV3 at 37°C.

(**a**) The 7Å resolution cryo-EM map. The E protein layer is colored in cyan and the glycosylation sites are colored in blue. Black triangle indicates an icosahedral asymmetric unit and the numbers represents icosahedral vertices. (**b**) Cross-section of a quarter of the cryo-EM map. (**c**) Superposition of cryo-EM structures of the mature DENV3 structures at 28°C (blue) and 37°C (red).

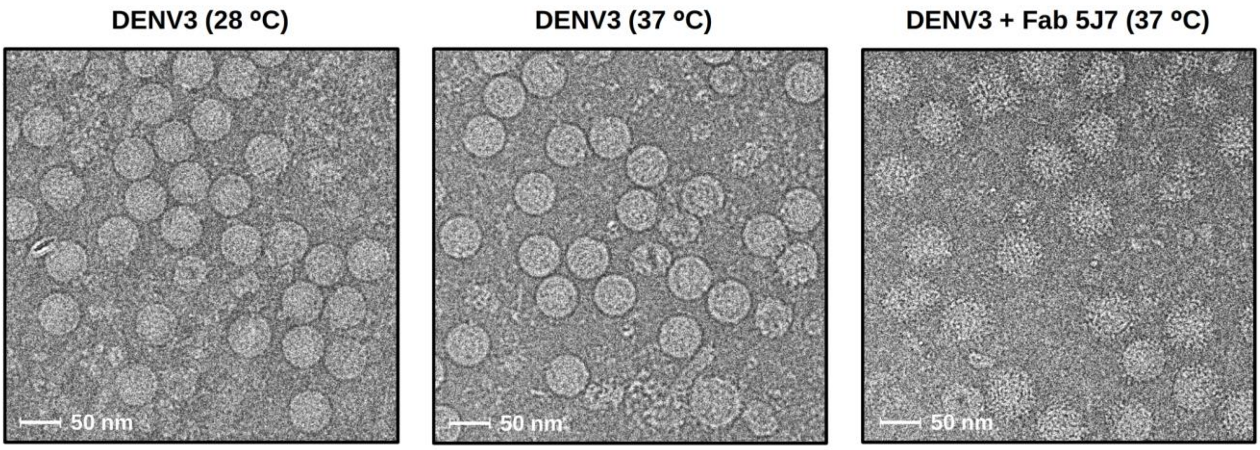

Supplementary Figure 4 Micrographs showing DENV3 controls at 28°C (left), 37°C (middle) and DENV3 complexed with Fab 5J7 at 37°C (right).

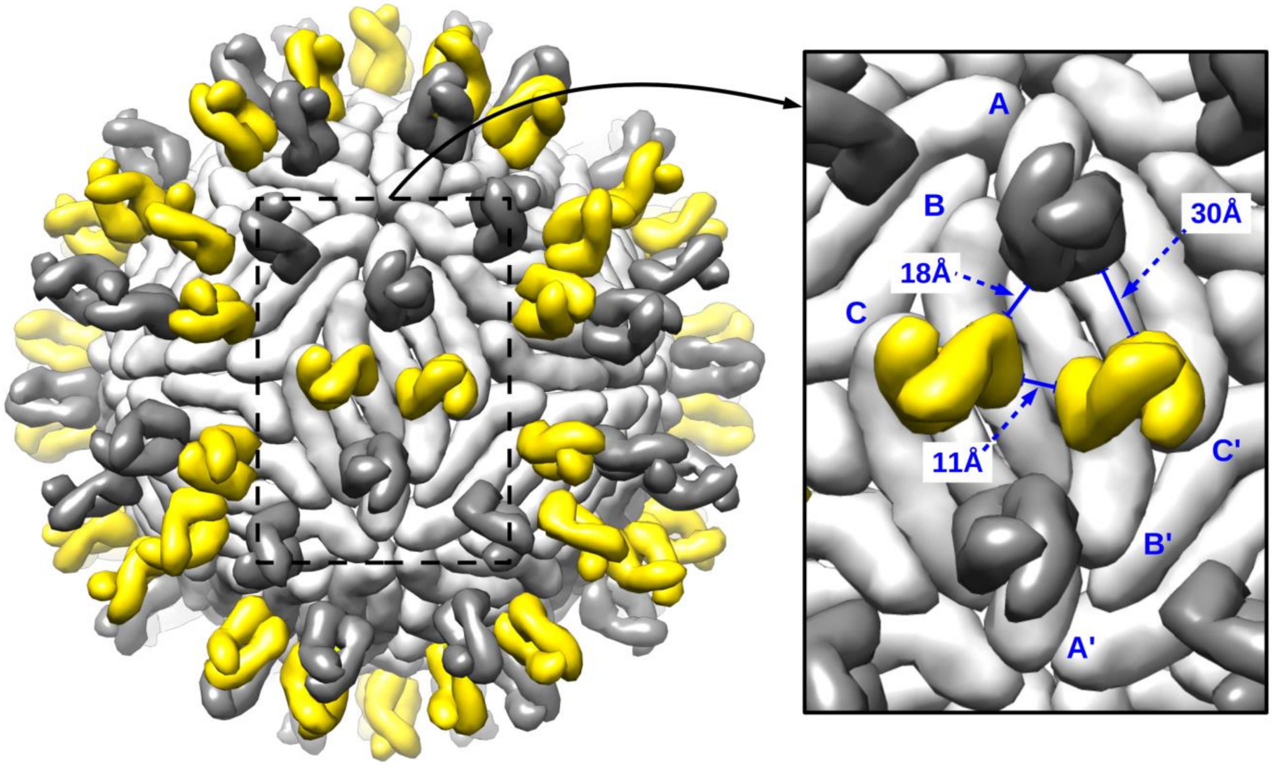

Supplementary Figure 5 Fabs bound to epitope on mols A, B and B′ will not interfere with the binding of Fabs to epitope to the second site (mols B, C, and A′). Superposition of the Fab (dark grey) bound to E protein epitope on mols A-B-B′ onto molecules B-C-A′ showed that the Fab molecule on mols A-B-B′ will not sterically prevent the Fab (yellow) from binding to molecules B-

C-A'. The closest distance between Fab molecules are shown as bars and the distances are indicated. E-proteins are colored in light grey and the three individual E proteins in an asymmetric unit are indicated.

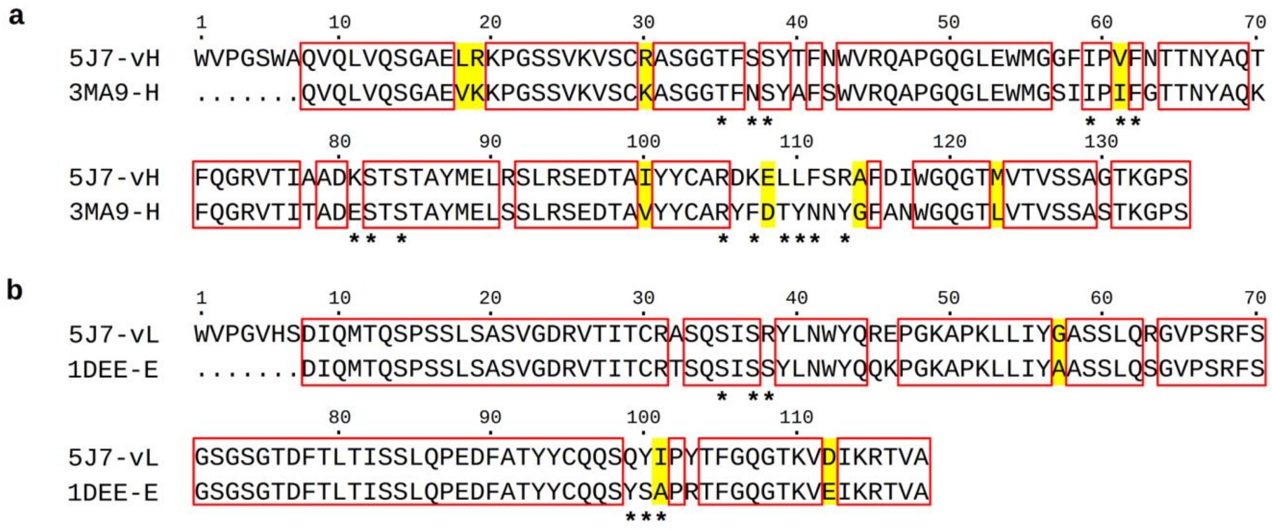

Supplementary Figure 6 Sequence alignment between the variable region of Fab 5J7 (heavy and light chains) and its corresponding starting homology models. (**a**) Sequence alignment between variable region of Fab 5J7 heavy chain and model 3MA9 chain H. (**b**) Sequence alignment between variable region of Fab 5J7 light chain and model 1DEE chain E. Conserved residues are boxed in red, residues with similar characteristics are boxed in yellow, CRDs are marked by "*". The first 7 N-terminal residues are not present in the starting homology models 3MA9-H and 1DEE-E.
